# Functional Identification of *Malus halliana MhbZIP23* Gene Demonstrates That It Enhances Saline–Alkali Stress Tolerance in *Arabidopsis thaliana*

**DOI:** 10.3390/plants13131803

**Published:** 2024-06-29

**Authors:** Wenqing Liu, Peng Li, Xiu Wang, Zhongxing Zhang, Yanxiu Wang

**Affiliations:** College of Horticulture, Gansu Agricultural University, Lanzhou 730070, China; 15720149915@163.com (W.L.); 19923288280@163.com (P.L.); 18409313974@163.com (X.W.); 18394133890@163.com (Z.Z.)

**Keywords:** *MhbZIP23*, saline–alkali stress, *Malus halliana*, functional characteristics

## Abstract

Saline–alkali stress is a significant abiotic stress that restricts plant growth globally. Basic region leucine zipper (*bZIP*) transcription factor proteins are widely involved in plants in response to abiotic stress such as saline–alkali stress. Based on transcriptome and quantitative real-time PCR (qRT-PCR), we found that the *MhbZIP23* gene could respond to saline–alkali stress. Despite this discovery, the underlying mechanism by which the *MhbZIP23* transcription factor responds to saline–alkaline stress remains unexplored. To address this gap in knowledge, we successfully cloned the *MhbZIP23* (MD05G1121500) gene from *Malus halliana* for heterologous expression in *Arabidopsis thaliana*, facilitating the investigation of its functional role in stress response. Compared to the wild type (WT), *Arabidopsis* plants demonstrated enhanced growth and a lower degree of wilting when subjected to saline–alkali stress. Furthermore, several physiological indices of the plants altered under such stress conditions. The transgenic *Arabidopsis* plants (OE-5, 6, and 8), which grew normally, exhibited a higher chlorophyll content and had greater root length in comparison to the control check (CK). *MhbZIP23* effectively regulated the levels of the osmoregulatory substance proline (Pro), enhanced the activities of antioxidant enzymes such as peroxidase (POD) and superoxide dismutase (SOD), and reduced the levels of malondialdehyde (MDA) and relative conductivity (REC). These actions improved the ability of plant cells in transgenic *Arabidopsis* to counteract ROS, as evidenced by the decreased accumulation of O_2_^−^ and hydrogen peroxide (H_2_O_2_). In summary, the *MhbZIP23* gene demonstrated effectiveness in alleviating saline–alkali stress in *M. halliana*, presenting itself as an outstanding resistance gene for apples to combat saline–alkali stress.

## 1. Introduction

The increasing severity of global soil salinization is significantly impacting the sustainable progress of contemporary agriculture [1]. Saline–alkali stress causes three types of damage to plant cells: osmotic stress, reactive oxygen species (ROS) imbalance, and ionic toxicity, with osmotic stress being the most common [2]. Osmotic stress and ionic toxicity may cause the accumulation of toxic compounds and nutrient uptake, resulting in the accumulation of ROS [3]. Excessive ROS buildup may cause membrane lipid peroxidation, leading to the loss of cellular hydration [4], ultimately severely damaging cellular structures and leading to a disturbance in metabolic pathways [5]. It is also possible to pass various plant transcription factors such as *DREB*, *WRKY*, *bZIP*, and *NAC* [6,7] to regulate gene expression so that the plant can adapt or mitigate the effects of saline–alkali stress, which enhances crop development and yield [8].

When plants are exposed to saline–alkali stress, a cascade of signals is produced to activate transcription factors. These factors then bind to specific regulatory elements [9], triggering RNA polymerase and transcription complexes to start transcribing and expressing particular genes [10]. Plants can also mediate signal transduction through various physiological pathways such as antioxidant enzymes, include superoxide dismutase SOD, POD, and catalase (CAT), as well as non-antioxidant enzymes such as H_2_O_2_ and MDA, effectively preventing oxidative stress [11]. The basic region leucine zipper (*bZIP*) family, known as one of the most extensive families of transcription factors linked to stress [10], plays a crucial part in overseeing plant development, growth, and response to abiotic stress [8]. The *Triticum aestivum* transcription factor *TabZIP60* was reported to improve tolerance to multiple abiotic stresses in transgenic *Arabidopsis* [12]; *Arabidopsis AtbZIP17* and *AtbZIP28* regulate root length during stress response [13]. Overexpression of *OsbZIP23* transgenic plants significantly increased drought tolerance, salt tolerance, and sensitivity to ABA in rice [14]. What is more interesting is that *bZIP* transcription factors at the level of post-translational modifications can similarly regulate saline–alkali stress. Kinases such as SnRK2 (Suc nonfermenting-1-related protein kinase 2) phosphorylate the *bZIP* proteins ABF2 and ABF3. These proteins then bind to the ABA response element (ABRE) in the promoter of specific genes, initiating downstream gene expression crucial for ABA signaling [15,16]. Additionally, *bZIP23*, a member of the *bZIP* transcription factor family [17], also plays a significant role in regulating abiotic stresses. However, little is known about the expression regulation mechanism of the *MhbZIP23* gene under saline–alkali stress in apple.

*M. halliana* is a widely used rootstock in the northwestern production areas of China [18], known for its strong resistance to saline–alkali stress and other adverse conditions [19]. In this study, we identified the *MhbZIP23* gene, which shows significant induction under saline–alkali stress, as evidenced by transcriptome analysis and qRT-PCR [20]. However, the regulatory mechanism of this gene under saline–alkali stress remains largely unexplored. Therefore, our study aimed to clone the gene and express it heterologously in *Arabidopsis* to characterize its function. Our goal is to identify the valuable resistance gene of *M. halliana* and establish a theoretical basis for selecting and breeding salt-resistant apple varieties.

## 2. Materials and Methods

### 2.1. Plant Materials and Treatments

The research on gene expression involved the utilization of *M. halliana* seedlings obtained from the Fruit Tree Tissue Culture Laboratory at the College of Horticulture, Gansu Agricultural University. The seedlings were first placed on rooting medium for 4 weeks and then transplanted into plastic pots (20.0 cm × 20.0 cm, with organic matter in a ratio of 3:1 with vermiculite) containing 5 plants each for consistent monitoring and nurturing. The apple rooting medium consisted of MS, 30 g/L sucrose, 8 g/L agar, 0.3 mg/L 6-BA, 0.2 mg/L IAA, and 0.1 mg/L GA_3_, pH 5.8–6.0.

Moreover, the WT *Arabidopsis* used is of the *Col-0* strain, which was conserved and provided by the Xiao-Fei Wang Laboratory of Shandong [21]. WT *Arabidopsis* plants were cultivated in MS medium (MS + 30 g/L sucrose + 8 g/L agar, pH = 5.8–6.0) and subjected to vernalization at 4 °C for 3 days before being transferred to a 26 °C incubator. Upon emergence of green seedlings, they were transplanted to a growth substrate composed of substrate and vermiculite in a 3:1 ratio. Inflorescences were utilized for *Arabidopsis* infestation during the blooming phase, with weekly sessions conducted 3–4 times. Transgenic *Arabidopsis* seeds were isolated on a Kanamycin-containing medium to maintain the purity of the transgenic plants [22]. The culture conditions included a temperature of 25 °C with a light cycle of 16 h/8 h. Each treatment was replicated three times with five plants each.

### 2.2. Saline–Alkali Stress Treatment of M. halliana Seedlings

For gene expression analysis, *M. halliana* seedlings with 8 robust and consistent leaves were selected and cultivated in 1/2 Hoagland solution for 15 days [23]. Following the cultivation period, saline–alkali stress was induced using a nutrient mix with 100 mM 1:1 NaCl: NaHCO_3_ (pH 8.0), and leaves were collected at 0 h, 6 h, 12 h, 24 h, 48 h, and 72 h. Each treatment was repeated three times, with five plants per replicate [24].

### 2.3. Bioinformatic Analysis of bZIP23 Gene

The apple *MhbZIP23* gene’s protein sequences were retrieved from the Phytozome database (https://phytozome-next.jgi.doe.gov/) (accessed on 10 October 2023). Basic physicochemical properties of these proteins were then predicted through ExPASy (https://web.expasy.org/protparam) (accessed on 8 April 2024). Online BLAST analyses were performed using the NCBI conserved domain database (https://www.ncbi.nlm.nih.gov/) (accessed on 8 April 2024), and motif predictions were performed using MEME (https://meme-suite.org/meme/) (accessed on 8 April 2024) [25,26,27]. The *MhbZIP23* phylogenetic tree was constructed using MEGAX (v11.0.13) (default parameters). Promoter *cis*-acting elements were analyzed using PlantCARE (https://bioinformatics.psb.ugent.be/webtools/plantcare/html/) (accessed on 10 April 2024) [28]. The *MhbZIP23* gene was examined for systematic clustering, gene structure, and conserved motifs through visualization and mapping with TBtools software (v2.096) [29].

### 2.4. Cloning and Expression Vector Construction of bZIP23 Gene

Total RNA was extracted from the samples using an RNA extraction kit from BioTeke Corporation in Beijing, China. Reverse transcription was carried out using TaKaRa’s PrimeScriptTM RT reagent Kit with gDNA Eraser (Perfect Real Time). Using DNAMAN software (v9.0), specific primers (Table 1) were designed for amplification by qRT-PCR after searching the apple genome database for the CDS sequence of *MhbZIP23*. The cDNA of *Malus halliana* plantlets was used as the template for qRT-PCR, with GAPDH serving as a reference. Quantitative data analysis was conducted using the 2^−ΔΔCt^ method, and differences were evaluated using Duncan’s test (*p* < 0.05) for one-way ANOVA. The PCR reaction program consisted of the following steps: 95 °C for 5 min, 95 °C for 30 s, 57 °C for 42 s, 72 °C for 100 s, and 72 °C for 10 min. The resulting PCR products underwent electrophoresis on an agarose gel for the purpose of isolating the desired genes. Subsequently, they were ligated into the pMD19-T cloning vector and transformed into *Escherichia coli* DH5α. Positive clones were identified and screened for sequencing accuracy. The accurately sequenced *MhbZIP23* plasmid was extracted and digested with *Sma I* and *Kpn I* restriction endonucleases for ligation into the pRI101 expression vector. Subsequently, screened bacteria were selected by the constitutively strong promoter 35S, which was introduced into *Escherichia coli*, and single positive colonies were identified and transferred to Agrobacterium GV3101 for genetic transformation using the freeze–thaw method [30]. Each sample was repeated three times.

### 2.5. Treatment and Screening of Genetically Modified Arabidopsis

Genetic modification was used to obtain transgenic *Arabidopsis* seeds with the *MhbZIP23* gene [31], and these seeds were screened on MS medium with 30 mg/L Kanamycin. Following this, the seeds were immersed in 75% ethanol for 5 min and then treated with 26% sodium hypochlorite for 10 min before being washed multiple times with ddH_2_O. The seeds were then placed on MS medium supplemented with 30 mg/L Kanamycin for cultivation. Resistant plants were identified through qRT-PCR amplification, resulting in the isolation of heterozygous transgenic *Arabidopsis*. To obtain pure transgenic *Arabidopsis* in the T3 generation, three generations were screened in total.

### 2.6. Transgenic Arabidopsis under Saline–Alkali Stress and Determination of Related Indicators

WT and T3 generation pure transgenic *Arabidopsis* seeds were aseptically treated before being inoculated on MS medium. They were subsequently incubated at 4 °C for 3 days. Afterwards, the seedlings were moved to MS and MS + 100 mmol/L of a NaCl and NaHCO_3_ mixture, respectively, where they were cultured in an incubator set to a constant temperature. Phenotypes and various indicators were observed and measured after 20 days.

For DAB staining, leaf samples were immersed in a 50 mM DAB solution for either 12 or 24 h before being decolorized in 95% ethanol until they turned white. For NBT staining, root tips or leaf samples were soaked in a 50 mM NBT solution for 4 h and subsequently decolorized in 95% ethanol until they appeared white. Chlorophyll content was measured as described by Cheng (2020) [32], and proline content was analyzed according to the method stated by Ferreira Junior (2018) [33]. MDA content was quantified using the thiobarbituric acid method [20]. The activities of SOD, POD, and CAT were determined spectrophotometrically with kits from Beijing Solarbio Science & Technology Co., Ltd. (Beijing, China). Relative conductivity was measured using the conductivity method (DDS-307) [34]. Each experiment was carried out in triplicate.

### 2.7. Data Analysis

In organizing the statistical data, Excel 2019 was utilized, while for data analysis, SPSS 21.0 was employed. Origin 2021 Pro was utilized to create visual representations, and the significance of the test data was analyzed using the least significant difference (LSD) test ANOVA method.

## 3. Results

### 3.1. Analysis of the MhbZIP23 Gene

**Figure 1:**
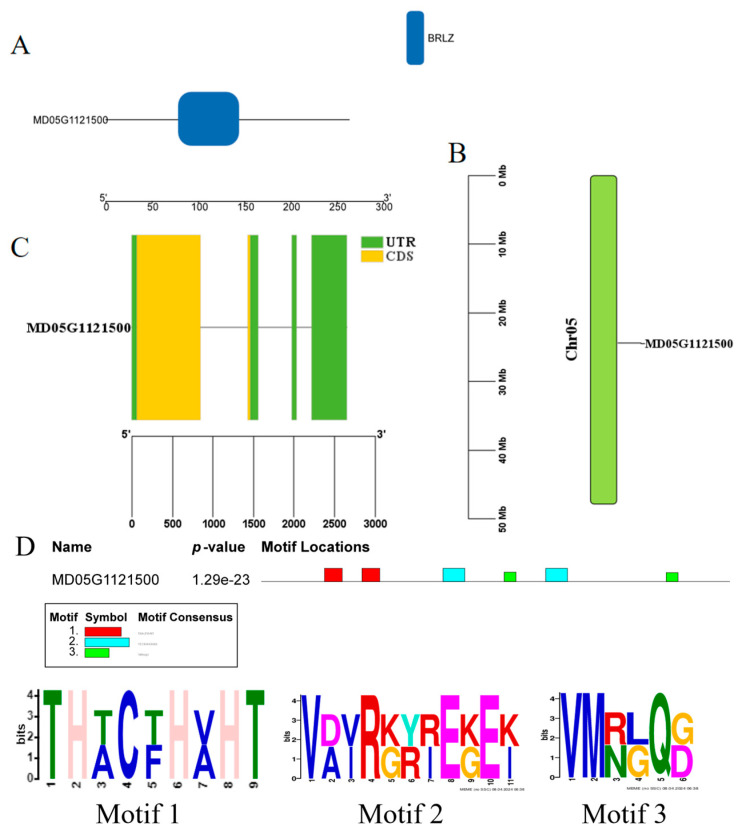
Basic physicochemical properties of apple *MhbZIP23* transcription factor: (**A**) domain; (**B**) chromosomal localization; (**C**) intron–exon; (**D**) conservative motif.

The encoding protein of *M. halliana MhbZIP23* (MD05G1121500) contains 263 amino acids, with a molecular weight of 28.48 kD, a theoretical isoelectric point of 6.18, and an acidic protein. In addition, it had an aliphatic index of 61.98, an instability index of 35.77, and a more stable protein with an average hydrophilicity of −0.617 (Table 2). This suggests that *MhbZIP23* is a more stable acidic hydrophilic protein. Subcellular localization revealed that the vast majority of *MhbZIP23* is located in the nucleus, with very few in the cell membrane. The structural domain prediction results showed that the amino acid sequence encoded by *MhbZIP23* has the structural features of *bZIP* family member, and the N-terminus contains a conserved BRLZ (the basic region leucin zipper domain) structural domain (Figure 1A). Chromosomal localization results showed that *MhbZIP23* was located on chromosome 5 (Figure 1B). The intron–exon results showed (Figure 1C) that the *MhbZIP23* gene contains four exons and three introns. The findings from the analysis of conserved motifs (Figure 1D) revealed that within the *M. halliana MhbZIP23* gene, there are three conserved motifs, namely, motif 1, motif 2, and motif 3. Notably, motif 1 corresponds to a leucine *bZIP* structural domain.

### 3.2. Protein Sequence Analysis of MhbZIP23 Gene

**Figure 2:**
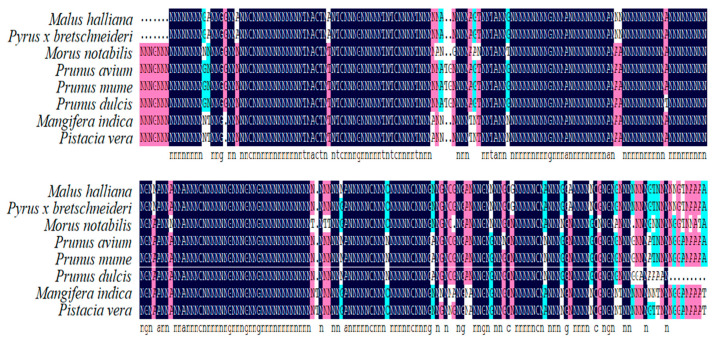
Protein sequence analysis of *MhbZIP23* gene and the protein of other species.

Comparison of the amino acid sequences of apple *MhbZIP23* with bZIP homologous proteins from other species, utilizing the NCBI database and DNAMAN software, revealed that *MhbZIP23* differed from other species’ proteins to some extent at the C-terminal end. However, the N-terminal end remained relatively conserved (Figure 2). Notably, the highest amino acid sequence homology was observed with *Pyrus × bretschneideri*.

### 3.3. Phylogenetic Tree Evolutionary Analysis of the Apple MhbZIP23 Protein

**Figure 3:**
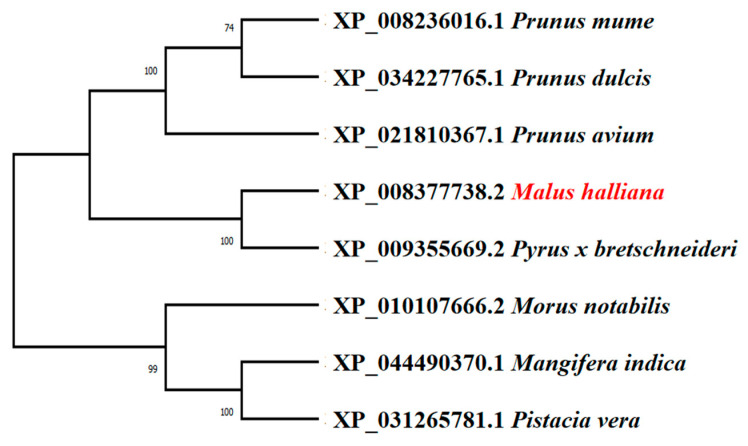
Phylogenetic analysis of *MhbZIP23* protein from *M. halliana* and other species.

A phylogenetic tree analysis of *bZIP23* proteins from different species was performed by MEGAX software according to the neighbor-joining method (Figure 3). The results show that *M. halliana* (XP_008377738.2) clustered with *Pyrus × bretschneideri* (XP_009355669.2) belong to the same subgroup. It is shown that *MhbZIP23* of *M. halliana* is the closest relative to *Pyrus × bretschneideri*, which predicts that it has similar biological functions.

### 3.4. Analysis of Cis-Acting Elements of MhbZIP23 Promoter

**Figure 4:**
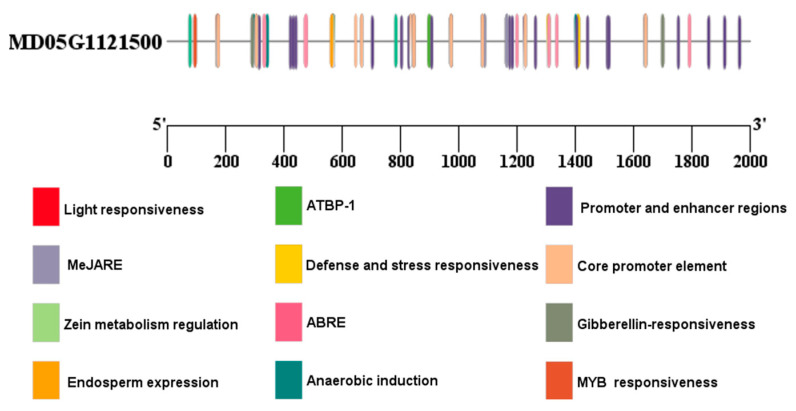
Analysis of *MhbZIP23* promoter *cis*-acting elements.

*Cis*-acting elements within the 2000 bp promoter region upstream of the gene play a crucial role in regulating gene expression. Examination of these components illuminates the regulatory mechanism of the *MhbZIP23* gene in apples, highlighting four primary categories: elements responsive to stress, elements responsive to hormones, elements responsive to plant development, and elements responsive to light (Figure 4). Stress response elements mainly include defense and stress responsiveness; hormone response elements mainly include ABRE elements, gibberellin response elements, etc.; plant development response mainly includes zein metabolism regulation; and light response elements include light responsiveness. These components suggest that the *MhbZIP23* gene may respond to the induction of different stresses such as saline–alkali, drought, and hormones.

### 3.5. Response of MhbZIP23 to Saline–Alkali Stress in M. halliana Seedlings

**Figure 5:**
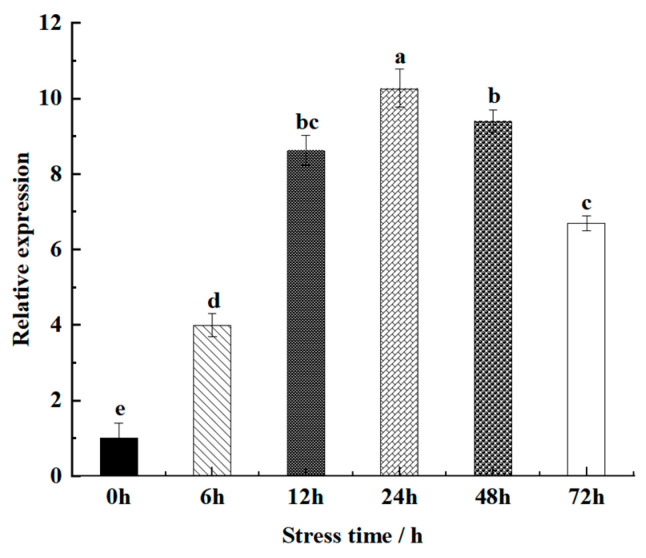
Expression levels of the *MhbZIP23* gene in *M. halliana* seedlings were measured under saline–alkali stress at 0, 6, 12, 24, 48, and 72 h. Note: Different letters above the bars indicate significant differences (*p* < 0.05) as assessed by one-way ANOVA and the LSD test (*p* < 0.05).

To investigate the gene expression pattern in *M. halliana* under saline–alkali stress at various time points, relative expression levels were determined using PCR fluorescence quantification. Figure 5 illustrates that the expression of *MhbZIP23* consistently increased compared to the control (0 h), peaking at 24 h with a 10.27-fold higher expression level than the control. These results suggest that *MhbZIP23* is responsive to saline–alkali stress.

### 3.6. Cloning of MhbZIP23 Gene

**Figure 6:**
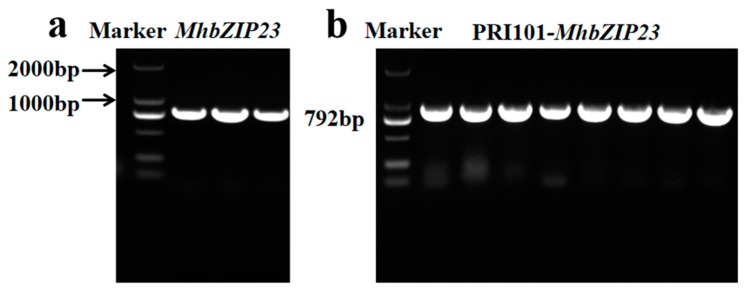
*MhbZIP23* gene amplification bands. (**a**) PCR product electrophoresis of cloned *MhbZIP23*. (**b**) PCR product electrophoresis of pRI101-*MhbZIP23*.

Total RNA of *M. halliana* leaves was extracted, and a 792 bp *MhbZIP23* target gene was obtained by qRT-PCR amplification (Figure 6). After purification and recovery of the target fragment, it was ligated with the pRI101 expression vector. Sequencing results showed that it was consistent with the base sequence of the *MhbZIP23* (XM_008379516) gene as compared to NCBI.

### 3.7. Screening and Identification of Transgenic Arabidopsis

**Figure 7:**
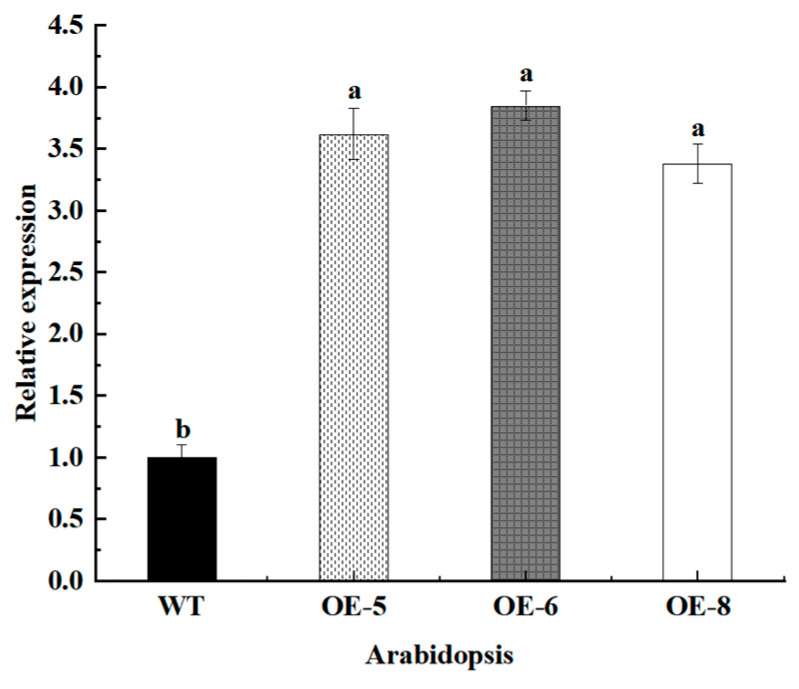
Identification of transgenic materials. Note: The values are the means ± standard errors, n = 3. Different lowercase letters indicate significant differences at the 0.05 level (*p* < 0.05).

The expression level of *MhbZIP23* in transgenic *Arabidopsis* was detected by qRT-PCR. Compared with wild-type plants, the expression level of *MhbZIP23* was higher in transgenic *Arabidopsis*, indicating that *MhbZIP23* was overexpressed in *Arabidopsis* lines (Figure 7).

### 3.8. Morphological Characteristics and Physiological Indicators of MhbZIP23 Transgenic Arabidopsis under Saline–Alkali Stress

**Figure 8:**
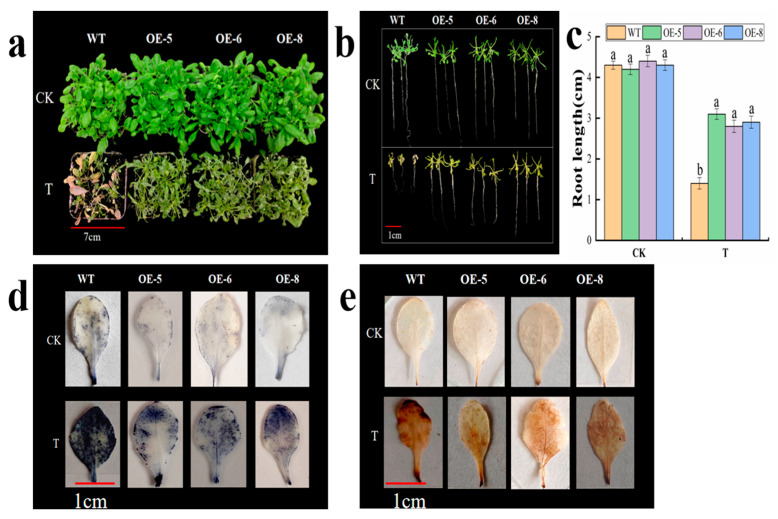
Phenotype, root length, NBT, and DAB staining of *MhbZIP23*-OE and WT *Arabidopsis* under normal conditions (CK) and saline–alkali stress (T): (**a**) phenotypes; (**b**) phenotypes of root length; (**c**) root length; (**d**) NBT staining; (**e**) DAB staining. Note: The values are the means ± standard errors, n = 3. Different lowercase letters indicate significant differences at the 0.05 level (*p* < 0.05).

**Figure 9:**
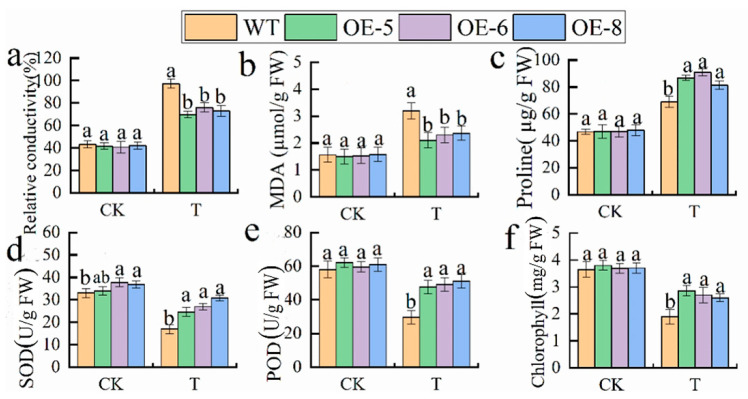
Physiological indices of *MhbZIP23*-OE and WT *Arabidopsis* under normal conditions (CK) and saline–alkali stress (T): (**a**) relative conductivity; (**b**) MDA content; (**c**) Pro content; (**d**) SOD activity; (**e**) POD activity; (**f**) chlorophyll content. Note: The values are the means ± standard errors, n = 3. Different lowercase letters indicate significant differences at the 0.05 level (*p* < 0.05).

To further examine the role of *M. halliana MhbZIP23* in response to saline–alkali stress (using a nutrient mix with 100 mM 1:1 NaCl:NaHCO_3_), we carried out saline–alkali tolerance assessments on WT and three transgenic *Arabidopsis* plants (OE-5, 6, and 8) with normal growth patterns. The findings from the experiments (Figure 8 and Figure 9) revealed that the transgenic *Arabidopsis* plants expressing *MhbZIP23* demonstrated enhanced resistance to saline–alkali stress when compared to WT plants. While there were no significant distinctions observed between OE lines and WT seedlings under regular conditions, both OE lines and WT seedlings exhibited leaf yellowing and wilting when exposed to saline–alkali stress in containers. Nonetheless, the OE lines displayed improved traits and higher levels of chlorophyll content in contrast to WT plants.

Moreover, different physiological parameters of the vegetation shifted when exposed to saline–alkali stress. While the root length of transgenic plants matches that of the WT under typical growing circumstances, it notably increases under saline–alkali stress in OE plants compared to the WT. DAB and NBT staining were performed on *Arabidopsis* plants, with darker blue and yellowish-brown shades indicating increased levels of O_2_^−^ and H_2_O_2_, respectively. There was no noticeable distinction between the OE strain and the WT under typical conditions. Nonetheless, when faced with saline–alkali stress, the leaf staining hue of the OE strain was significantly paler than that of the WT. This implies that the transgenic OE strain successfully lowered O_2_^−^ and H_2_O_2_ levels. Under saline–alkali stress, the OE line displayed reduced MDA content and REC in comparison to the WT. Conversely, significant increases were observed in the activities of SOD and POD, along with proline content, in the OE line over the WT. SOD and POD were elevated by 19.14–31.53% under saline–alkali stress in the transgenic lines compared to the WT. These observations suggest that the overexpression of the *MhbZIP23* gene enhances the antioxidant enzyme system’s performance in *Arabidopsis*. As a result, plant cells exhibit an improved ability to eliminate ROS, thereby decreasing the accumulation of O_2_^−^ and H_2_O_2_. This ultimately enhances the plant’s tolerance to saline–alkali conditions. 

## 4. Discussion

Saline–alkali stress is a critical abiotic factor that adversely impacts plant growth and development [35]. To cope with this challenging growing environment, plants have developed a variety of defense mechanisms for saline–alkali tolerance. These mechanisms involve stress-induced genes that encode numerous regulatory proteins, including protein kinases and transcription factors [36]. Transcription factors attach to the promoters of particular genomes responsive to stress to either trigger or hinder their expression, playing a critical part in the signal network for plant responses to saline–alkali stress [37]. Hence, uncovering genes that control plant resistance at a molecular level to enhance their resilience against saline–alkali stress holds significant research merit and promising practical implications [20].

The *bZIP* transcription factors are prevalent in eukaryotes [38] and have a crucial function in plant communication [39], a favorable reaction to environmental stresses like salt–alkaline pressure [40], stimulation of ABA [41], and other biochemical routes [42,43]. One instance is the broad triggering of *VvbZIP23* by environmental stresses such as drought, salt–alkali, and cold, aiding in the regulation of various stress reactions in *Vitis vinifera* [44]. *MdbZIP48*, *MdbZIP54* strongly responded to ABA and significantly increased the transcriptome level after plants were sprayed with ABA [45]. Moreover, the overexpression of *bZIP23* in rice regulates ABA biosynthesis and signaling, thereby improving drought and saline–alkali stress tolerance [46,47]. Thus, there is a broad influential role for *bZIP* transcription factors in plant abiotic stresses and their regulatory mechanisms [48]. The response mechanism of the *M. halliana MhbZIP23* transcription factor to saline–alkali stress is not yet fully understood in existing studies. Therefore, to explore its functional attributes under saline–alkali stress, we screened and cloned *MhbZIP23* (MD05G1121500) utilizing transcriptome data and qRT-PCR [49].

The results of the basic physicochemical properties of *MhbZIP23* indicated that *MhbZIP23* is a more stable acidic hydrophilic protein with the highest amino acid sequence homology to *Pyrus × bretschneideri*, which is inferred to be the closest relative [50]. We found the presence of several elements in the promoter region of the *MhbZIP23* gene, such as defense, stress responsiveness, and ABRE elements, suggesting that the *MhbZIP23* gene is capable of being activated to mediate ABA signaling, ultimately regulating saline–alkali tolerance [51,52].

During the experiment, *MhbZIP23* transgenic *Arabidopsis* plants were acquired, and their phenotypes were examined when exposed to saline–alkali conditions. Various parameters were assessed to gauge the effects of the stress. The transgenic *Arabidopsis* plants exhibited enhanced root elongation, increased vigor in growth, and elevated chlorophyll levels when subjected to saline–alkali stress. This indicates that the transgenic plants display improved resistance to saline–alkali conditions [23]. Additionally, *Arabidopsis* was stained with DAB and NBT to visually observe the accumulation of ROS in leaves [53]. The staining outcomes indicated no notable distinction in leaf hue between WT and transgenic OE plants in typical growth conditions. However, when subjected to saline–alkali stress, the color of WT plants was notably darker compared to the OE lines. These findings imply that the *MhbZIP23* gene may play a beneficial role in regulating saline–alkali stress responses in plants.

Plants experience a significant increase in ROS production when exposed to saline–alkali stress, triggering the production of antioxidant enzymes (POD, SOD, and CAT) to combat ROS buildup and shield the plant from oxidative injury [54]. The current study observed heightened levels of antioxidant enzyme activities: SOD and POD were elevated by 19.14–31.53% under saline–alkali stress in the transgenic plants compared to the WT. This indicates that transgenic plants enhance their antioxidant capacity by scavenging ROS, minimizing membrane destruction [55], and boosting antioxidant enzyme activities [56]. These outcomes are in line with the findings of Fang S (2021), who indicated that overexpression of *MsGSTU8* in transgenic tobacco resulted in decreased ROS accumulation and malondialdehyde levels, increased SOD, POD, and CAT activities, and improved resilience to saline–alkali stress [54,57]. MDA content and relative conductivity content were elevated in all plants under saline–alkali stress, but the OE plants were significantly lower than the WT [58], and the MDA activity could indirectly respond to the severity of the free radical attack [59], suggesting that all plants were damaged under saline–alkali stress, but obviously the transgenic plants were damaged less. Proline plays vital roles in enhancing plant resistance to various abiotic stresses [60]. The proline content in both the WT and genetically modified plants showed a significant increase when subjected to saline–alkali stress. However, the proline levels in the transgenic plants were considerably higher compared to those in the WT plants. This indicates that the genetically modified plants have the ability to efficiently modulate the levels of osmotic regulatory compounds, thereby mitigating oxidative harm to the plants [61]. These findings align with Wang Y’s (2018) research, which demonstrated that *MsWRKY11* overexpression led to increased proline levels, reduced ROS levels, and enhanced salt tolerance in soybean [62]. In this research, transgenic *Arabidopsis* exhibited a lesser extent of membrane damage when exposed to saline–alkali stress compared to WT *Arabidopsis*. This observation is supported by the decreased levels of electrolyte leakage and ROS and MDA accumulation, according to reference [56]. The findings suggest a strong association between the enhanced capability of the reactive oxygen species scavenging system and the increased saline–alkali resistance in plants overexpressing *MhbZIP23*. *MhbZIP23* augments saline–alkali resistance by activating the ROS scavenging mechanisms, thereby improving saline–alkali tolerance in *Arabidopsis*. This research shows the potential involvement of the *MhbZIP23* gene in regulating plant growth and development. Since growth and development processes are controlled by various pathways and signaling molecules, further examination of the role of the *MhbZIP23* gene in *M. halliana* growth and development can be conducted by cultivating the deletion mutant.

## 5. Conclusions

The *MhbZIP23* gene has been shown to positively respond to saline–alkali stress and enhance resistance in *Arabidopsis thaliana*. It plays a role in promoting chlorophyll synthesis, increasing antioxidant enzyme activity, and reducing membrane damage under saline–alkali stress conditions. Initial findings suggest that *MhbZIP23* enhances saline–alkali tolerance in *Arabidopsis thaliana* by scavenging reactive oxygen species, laying the groundwork for further exploration of its other functions.

## Figures and Tables

**Table 1:** Primers used in the experiment.

Primer	Sequence (5′-3′)
*MhbZIP23*-F	ATGGACGACCAGGAGGTGT
*MhbZIP23*-R	TCAGTTTGCTGCTGCGGC
*TYCZ*-*bZIP23*-SF	CATATGCCCGTCGACCCCGGGATGGACGACCAGGAGGTGT
*TYCZ*-*bZIP23*-SR	TCAGAATTCGGATCCGGTACCTCAGTTTGCTGCTGCGGC
*MhbZIP23*-*GFP*-F	GGACAGGGTACCCGGGGATCCATGGACGACCAGGAGGTGT
*MhbZIP23*-*GFP*-R	CACCATGGTACTAGTGTCGACGTTTGCTGCTGCGGCACG

**Table 2:** Basic information of *MdbZIP23* gene in apples.

Gene ID	Gene Name	Size/aa	MW/KD	pI	Aliphatic Index	Instability Index	Hydrophilicity
MD05G1121500	*MhbZIP23*	263	28.48	6.18	61.98	35.77	−0.617

## Data Availability

Data are contained within the article.
